# Mitochondrial replacement to prevent the transmission of mitochondrial DNA disease

**DOI:** 10.15252/embr.201540354

**Published:** 2015-04-17

**Authors:** Mary Herbert, Doug Turnbull

**Affiliations:** 1 Wellcome Trust Centre for Mitochondrial Research, Institute of Genetic Medicine, Newcastle UniversityNewcastle upon Tyne, UK; 2 Wellcome Trust Centre for Mitochondrial Research, Institute of Neuroscience, Newcastle UniversityNewcastle upon Tyne, UK

Mitochondrial medicine has seen major advances over recent years. The development of new genetic technology has enabled the genetic diagnosis to be established in an increasing number of patients. This has led to a recognition that mitochondrial diseases are not rare, but one of the most common group of genetic diseases. Associated with these advances, there have been undoubted improvements in the care of patients with mitochondrial disease. This is particularly true in the UK where the development of an NHS Highly Specialised service for patients with mitochondrial disease has enabled access of all mitochondrial patients to advanced diagnostic services, and to clinics which enable the multidisciplinary care for both children and adults.

Whilst all in the field would acknowledge the major achievements over the last few years, sadly the other advances have not been mirrored by advances in curative treatments 1. Whilst supportive care can make a major difference to a patient's life expectancy and quality of life, these remain progressive conditions, which lead to significant morbidity and mortality. In our focus groups with mitochondrial patients, whilst they greatly appreciate all the current efforts, for the vast majority of families with mitochondrial disease, the most important issue is how they can have healthy offspring. For families with nuclear genetic defects, the options are no different from other nuclear defects, but for women with mitochondrial DNA (mtDNA) disorders, the choices are considerably more challenging 2. It is upon this background we have to place the discussion about the ethics and feasibility of preventing the transmission of mitochondrial DNA disease by nuclear transfer techniques.

MtDNA is purely maternally inherited and since it is present in multiple copies, patients can either have all mutated mtDNA (homoplasmic mutations) or a mixture of mutated and wild-type mtDNA (heteroplasmic mutations). Women with mtDNA mutations already have a number of different options available. These range from not considering having any children through to preimplantation genetic diagnosis (PGD)—a technique which is very appropriate for some women with some heteroplasmic mtDNA defects 3. However, for women with either homoplasmic mtDNA mutations or with high levels of heteroplasmy, PGD is not an option. Thus, the approach of transferring nuclear DNA from an oocyte or zygote into a donor-enucleated oocyte or zygote to prevent the transmission of mtDNA disease is now a potentially viable option for mothers.

Despite the recent surge in interest in these approaches, it may come as a surprise that some of the proposed IVF techniques have been around since the early 1980s. The pioneering work of McGrath and Solter established that it is feasible to transfer the nuclear DNA (as pronuclei) between mouse zygotes 4 and that the offspring generated this way are healthy. The technique of pronuclear transfer has thus been used successfully in many different rodent laboratories for over 30 years. More recently, other options such as metaphase II spindle transfer 5 and polar body transfer 6 have been shown to be effective in either rodent or primate models. All methods of nuclear transfer have shown that the carryover of mtDNA between oocytes or zygotes is very low and well below the threshold of disease. Thus, in theory, it would be possible to prevent the transmission of mtDNA disease.

As with any scientific advance, it is never that easy. For mitochondrial replacement, there are four big challenges: ethics, legality, safety and efficacy. The ethical issues around this work are considerable and often polarise to either very opposed or very positive for the technique. These issues have been considered in great detail by the Nuffield Council on Bioethics (http://nuffieldbioethics.org/project/mitochondrial-dna-disorders/), but it is important to recognise that one's own ethical stance may influence the assessment of the whole technique. The legality and regulation of the technique will vary markedly between countries. In the UK, there is a highly regulated environment for IVF techniques and there has been very detailed consideration of the proposals to prevent transmission of mtDNA mutations including an extensive public enquiry which was broadly supportive of the technique. Following debates in which both Houses of Parliament voted overwhelmingly for a change to UK legislation, Regulations were passed to enable the Human Fertilisation and Embryology Authority (HFEA) to consider applications to use pronuclear transfer and spindle transfer in clinical treatment to prevent transmission of mtDNA disease. These Regulations will come into force in October 2015. Once the HFEA's requirements are known, clinics in the UK can then apply for a licence.

For patients, and the doctors looking after them, perhaps the biggest concern is the safety of these techniques and also how likely a woman is to become pregnant after the mitochondrial replacement. Both these issues have been extensively considered (studying both published and unpublished work) on three separate occasions by a panel of independent experts (including developmental biologists, IVF experts and clinical geneticists). On every occasion (including as recently as March 2014, http://www.hfea.gov.uk/8807.html), these independent experts have found no substantial reason to believe that these techniques would not be safe. They have suggested some further experiments to be completed before they think treatment licences should be considered, and they could see no reason why the legislation in the UK should not go forward.

It is rare that any new technique has undergone such extensive public and scientific debate, and of course, there will always be those opposed to such advances on either ethical or scientific grounds. However, we believe it is really important to remember why these techniques are being proposed and that ultimately we do hope that women with mtDNA disease will be able to make reproductive choices that include the possibility that their own offspring is free from disease.
